# Pseudo-differential operators and generalized random fields over tori

**DOI:** 10.1007/s11868-026-00840-9

**Published:** 2026-10-20

**Authors:** Nicolas Escobar

**Affiliations:** Department of Biostatistics, Epidemiology and Informatics, Perelman School of Medicine, University of Pennsylvania, Philadelphia, PA 19104 USA

**Keywords:** Matérn covariance, Gaussian random fields, Pseudo-differential operators on the torus, Variable-order symbols, Sample path regularity, Gaussian free field, 60G60, 35S05, 60G15, 60G17, 58J40, 62M40

## Abstract

Matérn covariance functions are ubiquitous in spatial statistics, valued for their interpretable parameters and well-understood sample path properties in Euclidean settings. This paper extends the theory of Matérn fields over tori through the pseudo-differential calculus. We first establish that a Matérn process on the *d*-dimensional torus has sample paths in $C^{\nu ^-}_{\textrm{loc}}$ for every ν>0 and no more, exactly as in the Euclidean case. Our main results concern symbols whose order varies with position. Given an order function $m \in C^\infty (\mathbb {T}^d;\mathbb {R})$ we construct a Gaussian field whose sample-path Hölder exponent is H(x)=m(x)-d/2 at each point, and we prove that the existence threshold m(x)>d/2 is local: the regularity of the field near a point depends on *m* only through its germ at that point. Finally we examine the canonical field, which supplies a direct check on both the threshold and the exponent. We use it to prove a rigidity statement: weighting the Matérn spectral density by $|k|^{-2}$ produces a superposition of ordinary Matérn fields of higher smoothness.

## Introduction

The analysis of spatial data on closed manifolds has become increasingly relevant across diverse scientific disciplines, from geospatial machine learning on the sphere [1] to neuroimaging on cortical surfaces [2, 3]. A predominant approach in this field involves modeling spatial phenomena as Gaussian random fields (GRFs), with the Matérn covariance function serving as the de facto standard for characterizing spatial dependence [4]. This methodological consensus is reinforced by the computational accessibility of tools such as the SPDE approach [4], which has seen extensive development over the past decade [5], and INLA (Integrated Nested Laplace Approximations) [6, 7].

We adopt the framework of pseudo-differential operators on tori. The torus serves as an ideal testing ground: it is geometrically non-trivial yet allows for explicit calculations. Moreover, tori are the standard setting for models with periodic boundary conditions—the Brillouin zone of a crystal lattice is a torus [8]—and in spatial statistics the circle $\mathbb {T}^1$ already carries a Matérn theory of its own [9].

Our first result is that a Matérn process on the *d*-dimensional torus with smoothness parameter ν>0 has sample paths in $C^{\nu ^-}_{loc}$, the same exponent as its Euclidean counterpart.

The situation of interest is that in which the symbol has no fixed order. Our main results concern fields built from symbols whose order varies with position. Such a field has a sample-path Hölder exponent H(x)=m(x)-d/2 that varies from point to point, and an existence threshold m(x)>d/2 that is local.

A third strand concerns spectral reweighting. Weighting the Matérn spectral density by the symbol of the inverse Laplacian produces a field of higher smoothness. We show that the resulting field decomposes as an explicit convergent superposition of ordinary Matérn fields of smoothnesses $\nu +1, \nu +2, \dots$.

This paper makes several contributions to the theory and practice of spatial modeling on manifolds. First, in Sect. 2, we establish the mathematical preliminaries, introducing the framework of pseudo-differential operators on tori and generalized random fields (GeRFs). This framework provides the necessary tools to rigorously analyze the relationship between spectral properties and sample path behavior. Section 3 settles the regularity of the stationary Matérn process on the torus, establishing the sharp exponent $C^{\nu ^-}_{loc}$. The argument uses only the order of the symbol.

Section 4 contains the main results. We define the variable-order Matérn field, compute its covariance, and prove a sharp two-sided pointwise regularity theorem together with a locality theorem showing that regularity is an invariant of the germ of the order function. Section 5 treats the canonical field. The appendix contains the kernel estimates on which Sects. 3 and 4 rest: the parametrix and pseudo-locality lemma for the variable-order class, the sample-path criterion of [10] in the form in which it is used, the local expansion of the Matérn kernel, a two-sided lattice estimate, and the periodisation bound used in Sect. 3 together with its truncation corollary.

## Preliminaries

### Notation, test functions and distributions

Throughout, $\mathbb {T}^d = \mathbb {R}^d/\mathbb {Z}^d$ carries period 1 and Lebesgue measure of total mass 1, and the Fourier coefficients of $u \in L^1(\mathbb {T}^d)$ are

$$ \hat{u}(k) \;=\; \int _{\mathbb {T}^d} u(x)\, e^{-2\pi i k\cdot x}\,dx, \qquad k \in \mathbb {Z}^d. $$

We write $\langle k\rangle = (1+|k|^2)^{1/2}$ and, for a fixed κ>0,

1 $$\begin{aligned} \lambda (k) \;:=\; \bigl (\kappa ^2 + 4\pi ^2|k|^2\bigr )^{1/2} , \end{aligned}$$

so that $\lambda (k) \asymp \langle k\rangle$. For a function *f* on $\mathbb {T}^d$ or on $\mathbb {Z}^d$ we write $\check{f}$ for its reflection, $\check{f}(x) = f(-x)$. All constants denoted *C* may change from line to line.

The space of test functions is $C^\infty (\mathbb {T}^d)$, topologised by the seminorms $u \mapsto \sup _{x}|\partial ^\beta u(x)|$. Since $\mathbb {T}^d$ is compact there is no decay condition to impose, and $u \in C^\infty (\mathbb {T}^d)$ if and only if $\sup _{k}\langle k\rangle ^{N}|\hat{u}(k)| < \infty$ for every $N \in \mathbb {N}$, the seminorms $u \mapsto \sup _k \langle k\rangle ^N |\hat{u}(k)|$ generating the same topology. We therefore write $\mathcal {S}(\mathbb {T}^d):= C^\infty (\mathbb {T}^d)$ and use the two descriptions interchangeably. Its dual $\mathcal {S}'(\mathbb {T}^d)$ is the space of distributions on $\mathbb {T}^d$; the transpose of the Fourier transform identifies $\mu \in \mathcal {S}'(\mathbb {T}^d)$ with the sequence $\hat{\mu }(k) = \langle \mu , e^{-2\pi i k\cdot }\rangle$.

Two classes of nonnegative sequences on $\mathbb {Z}^d$ will be needed. A positive measure μ on $\mathbb {Z}^d$ is *slowly growing*, written $\mu \in \mathcal {M}_{SG}^+(\mathbb {Z}^d)$, if

2 $$\begin{aligned} \sum _{k \in \mathbb {Z}^d} \langle k\rangle ^{-2N} \mu (\{k\}) \;<\; \infty \qquad \text {for some } N \in \mathbb {N}. \end{aligned}$$

These are the measures that extend to elements of $\mathcal {S}'(\mathbb {Z}^d)$, and hence the spectral measures of the random fields introduced below. Given (2), the estimate

$$ \bigl |\langle \mu ,\varphi \rangle \bigr | \;\le \; \Bigl (\sup _k \langle k\rangle ^{2N}|\varphi (k)|\Bigr ) \sum _{k}\langle k\rangle ^{-2N}\mu (\{k\}) $$

shows that $\varphi \mapsto \sum _k \varphi (k)\mu (\{k\})$ converges absolutely for every rapidly decreasing φ and is continuous on $\mathcal {S}(\mathbb {Z}^d)$; conversely, testing against $\varphi = \langle k\rangle ^{-2N}\textbf{1}_{\{|k| \le R\}}$ and letting $R \rightarrow \infty$ returns (2).

### Fourier multipliers and generalized random fields

Let $g: \mathbb {Z}^d \rightarrow \mathbb {C}$ be of polynomial growth—the *symbol* of the operator (3) below. Because multiplication by *g* preserves the sequences of rapid decrease, the prescription

3 $$\begin{aligned} \textrm{Op}(g)\,u \;:=\; \mathscr {F}^{-1}\bigl [g\,\hat{u}\bigr ], \qquad \text {that is,}\qquad \textrm{Op}(g)u(x) \;=\; \sum _{k\in \mathbb {Z}^d} g(k)\,\hat{u}(k)\, e^{2\pi i k\cdot x}, \end{aligned}$$

defines a continuous linear map $\mathcal {S}(\mathbb {T}^d) \rightarrow \mathcal {S}(\mathbb {T}^d)$. The operator $\textrm{Op}(g)$ extends to $\mathcal {S}'(\mathbb {T}^d)$ by $\langle \textrm{Op}(g)\mu , \varphi \rangle := \langle \mu , \textrm{Op}(\check{g})\varphi \rangle$, and the extension is continuous and agrees with (3) on $\mathcal {S}(\mathbb {T}^d)$. The operator maps real distributions to real distributions precisely when $\check{g} = \bar{g}$, and we assume this of every symbol below. Operators of the form (3) are the constant-order case of the quantization used in Sect. 4, where *g*(*k*) is replaced by a symbol *p*(*x*, *k*) depending on the base point; we use the notation $\textrm{Op}$ for both.

The operator we return to most often is the Matérn operator. Let $\mathcal {L}:= \kappa ^2 - \Delta$ with κ>0; its symbol is $\kappa ^2 + 4\pi ^2|k|^2 = \lambda (k)^2$, and for $\alpha \in \mathbb {R}$ we set

4 $$\begin{aligned} \mathcal {L}^{\alpha /2} \;:=\; \textrm{Op}\bigl (\lambda ^{\alpha }\bigr ) . \end{aligned}$$

The parameter α is the order of the operator; it is related to the Euclidean smoothness parameter ν of Sect. 3 by $\alpha = \nu + d/2$. The Schwartz kernel (Theorem A.2) of $\textrm{Op}(\lambda ^\alpha )$ is the distribution $\varrho _\alpha (x-y)$ with $\varrho _\alpha = \mathscr {F}^{-1}[\lambda ^\alpha ]$; it is a continuous function when α<-d, in which case the series $\varrho _\alpha (h) = \sum _{k}\lambda (k)^{\alpha }e^{2\pi i k\cdot h}$ converges absolutely, and only a distribution otherwise.

We now introduce the random objects.

#### Definition 2.1

*(Generalized random field)* A real generalized random field (GeRF) *Z* on $\mathbb {T}^d$ is a continuous linear map $\mathcal {S}(\mathbb {T}^d) \rightarrow L^2(\Omega ,\mathcal {A},\mathbb {P})$ taking real test functions to real random variables.

We write $\langle Z,\varphi \rangle$ for the random variable Z(φ). The field is *Gaussian* if $\langle Z,\varphi \rangle$ is a Gaussian random variable for every $\varphi \in \mathcal {S}(\mathbb {T}^d)$; linearity then makes every finite family $(\langle Z,\varphi _1\rangle ,\dots ,\langle Z,\varphi _n\rangle )$ jointly Gaussian, since a linear combination $\sum _i c_i\langle Z,\varphi _i\rangle$ equals $\langle Z, \sum _i c_i\varphi _i\rangle$. By the *law* of *Z* we mean the family of these finite-dimensional distributions or, equivalently, the measure induced on $\mathcal {S}'(\mathbb {T}^d)$ through Minlos’ theorem. We refer to [11] for this framework.

By the kernel theorem for tori (Theorem A.2) a GeRF has a mean distribution $m_Z$ and a covariance distribution $C_Z \in \mathcal {S}'(\mathbb {T}^d\times \mathbb {T}^d)$, the latter characterised by

5 $$\begin{aligned} \mathbb {C}\textrm{ov}\bigl (\langle Z,\varphi \rangle , \langle Z,\phi \rangle \bigr ) \;=\; \langle C_Z, \varphi \otimes \bar{\phi }\rangle , \qquad \varphi ,\phi \in \mathcal {S}(\mathbb {T}^d) . \end{aligned}$$

We assume $m_Z = 0$ throughout. The field *Z* is *stationary* if there is a distribution $\rho _Z \in \mathcal {S}'(\mathbb {T}^d)$ with

6 $$\begin{aligned} \langle C_Z, \varphi \otimes \bar{\phi }\rangle \;=\; \langle \rho _Z, \varphi *\check{\bar{\phi }}\rangle , \end{aligned}$$

and $\rho _Z$ is then positive definite. By the Bochner–Schwartz theorem in the form valid on locally compact abelian groups (Theorem A.3) there exists a unique $\mu _Z \in \mathcal {M}^+_{SG}(\mathbb {Z}^d)$, the *spectral measure* of *Z*, with $\rho _Z = \mathscr {F}^{-1}(\mu _Z)$, equivalently $\mu _Z(\{k\}) = \hat{\rho }_Z(k)$; when *Z* is Gaussian, $\mu _Z$ determines its law.

Two GeRFs are said to be equal *in second order*, written $Z {\mathop {=}\limits ^{\text {2nd o.}}}Z'$, if they have the same mean and covariance distributions; for Gaussian fields this is equality in law. The notation also fixes the sense in which the equations below are solved: $\textrm{Op}(g)U {\mathop {=}\limits ^{\text {2nd o.}}}X$ asserts that the field $\textrm{Op}(g)U$ has the first two moments of *X*. With this convention, if *U* is stationary with spectral measure $\mu _U$ then $\textrm{Op}(g)U$ is stationary with spectral measure $|g|^2\mu _U$ (Proposition A.7), and the solvability of $\textrm{Op}(g)U {\mathop {=}\limits ^{\text {2nd o.}}}X$ is settled by Theorem A.8.

The white noise *W* on $\mathbb {T}^d$ (Definition A.10) is the stationary GeRF with $\rho _W = \delta _0$ and spectral measure $\mu _W(\{k\}) = 1$ for every $k \in \mathbb {Z}^d$ (Proposition A.11). The Matérn field of Sect. 3 is the stationary solution of

7 $$\begin{aligned} \mathcal {L}^{\alpha /2} u \;{\mathop {=}\limits ^{\text {2nd o.}}}\; W , \qquad \alpha = \nu + \tfrac{d}{2} , \end{aligned}$$

with spectral measure $\lambda (k)^{-2\alpha }$.

## Regularity of Matérn processes on tori

Sample path regularity is measured by determining whether paths belong to Hölder spaces.

### Definition 3.1

*(Local Hölder and almost-Hölder spaces)* Let $n \in \mathbb {N}_0$ and $\gamma \in [0,1]$. Let $\mathcal {O}\subseteq \mathbb {T}^d$ be an open set.

1. The local Hölder space $C^{n,\gamma }_{loc}(\mathcal {O})$ is the space of functions *f* on $\mathcal {O}$ for which $\partial ^{\alpha } f$ exists for all multi-indices $\alpha \in \mathbb {N}_0^d$ with $|\alpha | \le n$, and such that the highest order partial derivatives satisfy a Hölder condition of the form: for all compact $K \subset \mathcal {O}$ there is $C_K>0$ with
  $$\begin{aligned} |\partial ^{\alpha } f(x) -\partial ^{\alpha } f(y)| \le C_K\Vert x-y\Vert ^\gamma \end{aligned}$$
  for all x,y∈K and |α|=n.
2. The local almost-Hölder space $C^{(n+\gamma )^-}_{loc}(\mathcal {O})$ is $\bigcap _{n'+\gamma '<n+\gamma }C^{n',\gamma '}_{loc}(\mathcal {O})$.

Every regularity statement in this paper is obtained from a criterion, which characterises membership in the almost-Hölder scale by the size of the second-order increment of the covariance kernel across the diagonal. For a kernel *k* on $\mathcal {O}\times \mathcal {O}$, multi-indices $\alpha ,\beta$ and $h \in \mathbb {R}^d$ small, write

8 $$\begin{aligned} \square ^{\alpha ,\beta }_h k(x) \;:=\; \partial ^{\alpha ,\beta }k(x+h,x+h) - \partial ^{\alpha ,\beta }k(x+h,x) - \partial ^{\alpha ,\beta }k(x,x+h) + \partial ^{\alpha ,\beta }k(x,x) , \end{aligned}$$

where $\partial ^{\alpha ,\beta }$ differentiates α times in the first argument and β times in the second. For $\alpha =\beta =0$ this is the variogram, $\square _h k(x) = \mathbb {E}|f(x+h)-f(x)|^2$.

### Theorem 3.2

(Sample-path Hölder criterion; [10, Theorem 3.1]) Let $n \in \mathbb {N}_0$, $\gamma \in (0,1]$, let $\mathcal {O}\subseteq \mathbb {R}^d$ be open and let $f \sim \mathcal{G}\mathcal{P}(0,k)$ be a centred Gaussian process on $\mathcal {O}$. Then *f* has samples in $C^{(n+\gamma )^-}_{loc}(\mathcal {O})$ if and only if $k \in C^{n\otimes n}(\mathcal {O}\times \mathcal {O})$ and

9 $$\begin{aligned} \bigl |\square ^{\alpha ,\beta }_h k(x)\bigr | \;=\; \mathcal {O}\bigl (\Vert h\Vert ^{2\epsilon }\bigr ) \qquad (h \rightarrow 0), \end{aligned}$$

locally uniformly in *x*, for all $\epsilon \in (0,\gamma )$ and all multi-indices with $|\alpha | = |\beta | = n$. When k(x,y)=ρ(x-y) is stationary the conditions read $\rho \in C^{2n}(\mathbb {R}^d)$ and

10 $$\begin{aligned} \bigl |\partial ^{\alpha }\rho (h) - \partial ^{\alpha }\rho (0)\bigr | \;=\; \mathcal {O}\bigl (\Vert h\Vert ^{2\epsilon }\bigr ) \qquad (h\rightarrow 0) \end{aligned}$$

for all $\epsilon \in (0,\gamma )$ and all α with |α|=2n.

Both the criterion and the definition of the almost-Hölder scale are local, so they apply on a manifold chart by chart; this is the reading of [10, Sec. 3.7], and on $\mathbb {T}^d$, which is locally isometric to $\mathbb {R}^d$.

For Euclidean Matérn processes, [10, Proposition 3.1] deduces from the isotropic case of Theorem 3.2, not reproduced here, the following benchmark.

### Proposition 3.3

([10, Proposition 3.1]) A centered Matérn GP on $\mathbb {R}^d$ with smoothness parameter ν>0 has samples in $C^{\nu ^-}_{loc}(\mathcal {O})$ and no more.

The natural question is whether this extends to processes over tori. The torus Matérn covariance is the periodisation of its Euclidean counterpart,

11 $$\begin{aligned} k_\nu (x,x') = c_{\nu ,\sigma } \sum _{n \in \mathbb {Z}^d} \left( \frac{\sqrt{2\nu }\,\Vert x - x' + n\Vert }{\ell }\right) ^\nu K_\nu \left( \frac{\sqrt{2\nu }\,\Vert x - x' + n\Vert }{\ell }\right) , \end{aligned}$$

where ℓ>0 is the range, $c_{\nu ,\sigma } = \sigma ^2 2^{1-\nu }/\Gamma (\nu )$ is the usual normalisation, which plays no role in what follows, and $\kappa = \sqrt{2\nu }/\ell$ is the inverse range appearing in (1).

### Proposition 3.4

(Path regularity of torus Matérn processes) A GP over $\mathbb {T}^d$ with covariance (11) and smoothness parameter ν>0 has samples in $C^{\nu ^-}_{loc}(\mathcal {O})$ and no more. The exponent coincides with the Euclidean one of Proposition 3.3.

### Remark 3.5

The proof is deferred to Appendix A.7. The point of the proof we offer is its use of the pseudo-differential theory. There are simpler proofs by alternative methods.

### Remark 3.6

(Two conventions) Two objects are called the Matérn field on $\mathbb {T}^d$. The periodisation (11) has symbol of order -2ν-d; the SPDE field $\mathcal {L}^{\alpha /2}u {\mathop {=}\limits ^{\text {2nd o.}}}W$ has spectral measure of order -2α. They coincide when $\alpha = \nu + d/2$, the convention of [4], which is the one fixed for (4) and used throughout.

## Fields of variable order and locally prescribed regularity

So far we have considered symbols that do not depend on the base point. For such symbols the operator is a Fourier multiplier, and the pseudo-differential calculus, while convenient, is not indispensable. This section develops the setting of symbols σ(x,k) whose order varies with *x*.

### Variable-order symbols on the torus

#### Definition 4.1

*(Variable-order symbol class)* Let $m \in C^\infty (\mathbb {T}^d;\mathbb {R})$ and $0 \le \delta < 1$. A function $p: \mathbb {T}^d \times \mathbb {Z}^d \rightarrow \mathbb {C}$ belongs to $S^{m(\cdot )}_{1,\delta }(\mathbb {T}^d\times \mathbb {Z}^d)$ if for all multi-indices $\alpha ,\beta \in \mathbb {N}_0^d$ there is $C_{\alpha \beta }>0$ with

$$ \bigl |\Delta _k^\alpha \partial _x^\beta \, p(x,k)\bigr | \;\le \; C_{\alpha \beta }\,\langle k\rangle ^{\,m(x) - |\alpha | + \delta |\beta |}, \qquad (x,k) \in \mathbb {T}^d\times \mathbb {Z}^d, $$

where $\langle k\rangle = (1+|k|^2)^{1/2}$ and $\Delta _k^\alpha = \Delta _1^{\alpha _1}\cdots \Delta _d^{\alpha _d}$ is the forward difference operator on $\mathbb {Z}^d$, built from $\Delta _j f(k) = f(k+e_j) - f(k)$ with $e_j$ the *j*-th standard basis vector. The operator defined as

$$ \textrm{Op}(p)f(x) \;=\; \sum _{k\in \mathbb {Z}^d} p(x,k)\,\hat{f}(k)\, e^{2\pi i k\cdot x} $$

is called the quantization of *p* (see [12, 13]). We call *p* *elliptic of variable order m* if there are c,C>0 and $k_0$ with $c\langle k\rangle ^{m(x)} \le |p(x,k)| \le C\langle k\rangle ^{m(x)}$ for $|k|\ge k_0$.

#### Lemma 4.2

(The canonical variable-order symbol) With λ as in (1), $q_m(x,k):= \lambda (k)^{-m(x)}$ lies in $S^{-m(\cdot )}_{1,\delta }(\mathbb {T}^d\times \mathbb {Z}^d)$ for every δ>0, and lies in $S^{-m(\cdot )}_{1,0}$ if and only if *m* is constant.

#### Proof

By the chain rule, $\partial _x^\beta \lambda (k)^{-m(x)} = \lambda (k)^{-m(x)}\, P_\beta \!\bigl (\partial ^{\gamma }m(x)\bigr )_{\gamma \le \beta }\, \bigl (\log \lambda (k)\bigr )^{|\beta |}$ for a polynomial $P_\beta$, and $(\log \lambda (k))^{|\beta |} \le C_{\beta ,\delta }\,\lambda (k)^{\delta |\beta |}$ for every δ>0. Difference operators in *k* gain a full power of $\langle k\rangle ^{-1}$. Conversely, if $\partial _j m(x_*)\ne 0$ then $|\partial _{x_j} q_m(x_*,k)| = |\partial _j m(x_*)|\,\lambda (k)^{-m(x_*)}\log \lambda (k)$, which is not $O(\lambda (k)^{-m(x_*)})$. □

### The variable-order Matérn field

#### Definition 4.3

*(Variable-order Matérn field)* Let $m\in C^\infty (\mathbb {T}^d;\mathbb {R})$, κ>0, and let *W* be white noise on $\mathbb {T}^d$ (Definition A.10). The *variable-order Matérn field with order function m* is the GeRF

$$ u \;=\; \textrm{Op}(q_m)\,W, \qquad q_m(x,k) = \lambda (k)^{-m(x)}. $$

Because $q_m$ is a symbol of finite order and *W* is a GeRF, *u* is a well-defined GeRF on all of $\mathbb {T}^d$ for every real-valued *m*: the adjoint $\textrm{Op}(q_m)^*$ maps $\mathcal {S}(\mathbb {T}^d)$ into itself, so $\langle u,\varphi \rangle := \langle W, \textrm{Op}(q_m)^*\varphi \rangle$ is defined for all test functions.

#### Proposition 4.4

The covariance kernel of *u* is

12 $$\begin{aligned} C_u(x,y) \;=\; \sum _{k\in \mathbb {Z}^d} \lambda (k)^{-\left( m(x)+m(y)\right) } \, e^{2\pi i k\cdot (x-y)} , \end{aligned}$$

which is positive semi-definite for every real-valued *m*, and finite on the diagonal at *x* if and only if m(x)>d/2. When $m \equiv \nu + d/2$ is constant, (12) is the Whittle–Matérn covariance of Sect. 3 with smoothness ν.

#### Proof

The identity is immediate from $\mathbb {E}[\hat{W}(k)\overline{\hat{W}(k')}]=\delta _{kk'}$, which is Proposition A.6 applied to *W* together with $\mu _W \equiv 1$ (Proposition A.11). Positive semi-definiteness follows: setting $\phi (x):= \bigl (\lambda (k)^{-m(x)} e^{2\pi i k\cdot x}\bigr )_{k\in \mathbb {Z}^d}$, we have $C_u(x,y) = \langle \phi (x),\phi (y)\rangle _{\ell ^2}$ whenever the series converges, and in general $C_u$ is the kernel of $\textrm{Op}(q_m)\textrm{Op}(q_m)^*$. The diagonal is $C_u(x,x) = \sum _k \lambda (k)^{-2m(x)}$, finite iff 2m(x)>d. □

### Pointwise regularity

Throughout, $$U:= \{x\in \mathbb {T}^d: m(x) > d/2\}$$ and

$$ H(x) \;:=\; m(x) - \tfrac{d}{2}, $$

and for an open V⊆U we write $\gamma _V:= \inf _V H$.

#### Theorem 4.5

(Pointwise regularity and the local threshold) Let *u* be as in Definition 4.3.

(i) (Upper bound.) *u* has a modification whose restriction to *U* is continuous and which, for every open *V* with $\overline{V} \subset U$, lies in $C^{\gamma _V^-}_{loc}(V)$ almost surely.
(ii) (Sharpness.) For every open V⊆U and every $\gamma > \gamma _V$, almost surely $u|_V \notin C^{\gamma ^-}_{loc}(V)$.
(iii) (Below threshold.) If *V* is open with $\sup _V m < d/2$, then almost surely $u|_V \notin L^2(V)$: on *V* the field is a genuine distribution, not a function.

Consequently, *m* being continuous, for every $x_0 \in U$

$$ \sup \bigl \{\gamma >0 \;:\; u \in C^{\gamma ^-}_{loc}(V) \text { a.s.\ for some neighbourhood } V \ni x_0 \bigr \} \;=\; H(x_0), $$

so the sample-path exponent of *u* at $x_0$ is $H(x_0)$.

#### Proof

Write h=x-y, $x_0 = \tfrac{1}{2}(x+y)$ and $\Lambda (k) = \log \lambda (k)$. Expanding $C_u(x,x) - 2C_u(x,y) + C_u(y,y)$ using (12),

13 $$\begin{aligned} \begin{aligned} \mathbb {E}\bigl |u(x)-u(y)\bigr |^2 \;=\;&\underbrace{\sum _{k}\Bigl (e^{-m(x)\Lambda (k)} - e^{-m(y)\Lambda (k)}\Bigr )^{\!2}}_{\mathrm {(I)}} \\&\;+\; \underbrace{2\sum _{k} e^{-(m(x)+m(y))\Lambda (k)}\bigl (1-\cos 2\pi k\cdot h\bigr )}_{\mathrm {(II)}} . \end{aligned} \end{aligned}$$

Factoring out the midpoint value $e^{-\frac{1}{2}(m(x)+m(y))\Lambda }$ from the bracket and using $a - b = \sqrt{ab}\,\bigl (\sqrt{a/b}-\sqrt{b/a}\bigr ) = 2\sqrt{ab}\sinh \bigl ( \tfrac{1}{2}\log (a/b)\bigr )$ with $a = e^{-m(x)\Lambda }$, $b = e^{-m(y)\Lambda }$,

$$ \mathrm {(I)} \;=\; 4\sum _k e^{-(m(x)+m(y))\Lambda } \sinh ^2\!\bigl (\tfrac{1}{2}(m(x)-m(y))\Lambda \bigr ). $$

Since $$|m(x)-m(y)| \le \Vert \nabla m\Vert _\infty |h|$$ and $$\sinh ^2 t \le t^2 e^{2|t|}$$,

$$ \mathrm {(I)} \;\le \; C|h|^2 \sum _k \Lambda (k)^2\, \lambda (k)^{-2\,m(x_0) + C'|h|}. $$

For $x_0$ in a compact subset of *U* and |*h*| small the exponent satisfies $2m(x_0)-C'|h| > d + \eta$ for some η>0, so the sum converges and $\mathrm {(I)} = O(|h|^2)$, locally uniformly.

Put $\sigma := \tfrac{1}{2}\bigl (m(x)+m(y)\bigr )$, so that $\mathrm {(II)} = 2\sum _k \lambda (k)^{-2\sigma }(1-\cos 2\pi k\cdot h)$ is exactly the sum of Lemma A.17 with weight w≡1 and s=σ. Because $x_0$ is the midpoint, the first-order terms cancel and $\sigma = m(x_0) + O(|h|^2)$. Suppose first $d/2< m(x_0) < d/2+1$. Then σ stays in a compact subinterval of (d/2,d/2+1) for $x_0$ in a compact subset of *U* on which H<1 and for |*h*| small, so Lemma A.17(a) applies with constants uniform in σ and gives

$$ c\,|h|^{2\sigma -d} \;\le \; \mathrm {(II)} \;\le \; C\,|h|^{2\sigma -d}, $$

and

$$ |h|^{2\sigma -d} \;=\; |h|^{2\,H(x_0)}\,|h|^{O(|h|^2)} \;=\; |h|^{2\,H(x_0)}\bigl (1+o(1)\bigr ), $$

locally uniformly near $x_0$. Since $2H(x_0) < 2$ in this range, (II) dominates (I) and (13) is two-sided of order $|h|^{2H(x_0)}$. If instead $m(x_0) = d/2+1$, i.e. $H(x_0)=1$, part (b) of the same lemma gives the upper bound $\mathrm {(II)} = \mathcal {O}(|h|^{2\epsilon })$ for every ϵ<1, which is all the “if” half of Theorem 3.2 requires.

In the notation (8) with n=0 we have $\square _h C_u(x) = \mathbb {E}|u(x+h)-u(x)|^2$. Let *V* be open with $\overline{V}\subset U$ and $\gamma _V\le 1$. The upper bound above holds uniformly on compact subsets of *V* and gives $\square _h C_u(x) = \mathcal {O}(\Vert h\Vert ^{2\epsilon })$ for every $\epsilon <\gamma _V$, so (9) holds with n=0 and the “if” half of Theorem 3.2 yields (i). For (ii), let $\gamma >\gamma _V$ and choose $x_1 \in V$ with $H(x_1)<\min (\gamma ,1)$ together with $\epsilon \in (H(x_1),\min (\gamma ,1))$. The lower bound gives $\square _h C_u(x_1) \ge c\Vert h\Vert ^{2H(x_1)}$, which is not $\mathcal {O}(\Vert h\Vert ^{2\epsilon })$; by the “only if” half of Theorem 3.2, $u|_V \notin C^{\gamma ^-}_{loc}(V)$. Such an $x_1$ exists whenever $\gamma _V<1$. If $\gamma _V=1$ and the infimum is attained on an open set—equivalently, if H≡1 on some open $V_1\subseteq V$—then by (12) the restriction of $C_u$ to $V_1\times V_1$ depends on x-y alone and equals the stationary Matérn covariance with $s=m|_{V_1}$, so Proposition 3.4 applies on $V_1$. The same observation covers every point at which *m* is locally constant.

Differentiating the defining series of Definition 4.1 term by term gives the Leibniz identity

14 $$\begin{aligned} \partial _{x_j}\textrm{Op}(q_m) \;=\; \textrm{Op}\bigl (2\pi i k_j\, q_m\bigr ) \;+\; \textrm{Op}\bigl (\partial _{x_j} q_m\bigr ) . \end{aligned}$$

Here $2\pi ik_j q_m \in S^{-m(\cdot )+1}_{1,\delta }$, while by Lemma 4.2

$$ \partial _{x_j} q_m(x,k) \;=\; -\,\partial _j m(x)\,\log \lambda (k)\; \lambda (k)^{-m(x)} \;\in \; S^{-m(\cdot )+\delta }_{1,\delta }, $$

so the second term of (14) is lower in order by 1-δ>0. Iterating (14),

15 $$\begin{aligned} \partial ^\alpha \textrm{Op}(q_m) \;=\; \textrm{Op}\bigl ((2\pi i k)^{\alpha } q_m\bigr ) \;+\; E_\alpha , \qquad E_\alpha \in \textrm{Op}\,S^{-m(\cdot )+|\alpha |-(1-\delta )}_{1,\delta } . \end{aligned}$$

The leading term factors as $(2\pi i k)^{\alpha }q_m(x,k) = \theta _\alpha (k)\,q_{m-|\alpha |}(x,k)$, where

16 $$\begin{aligned} \theta _\alpha (k) \;:=\; (2\pi i k)^{\alpha }\lambda (k)^{-|\alpha |} \end{aligned}$$

is an *x*-independent multiplier with $|\theta _\alpha | \le (2\pi )^{|\alpha |}$ and, by the multinomial theorem,

17 $$\begin{aligned} \sum _{|\alpha |=n}\frac{n!}{\alpha !}\,|\theta _\alpha (k)|^2 \;=\; \frac{(2\pi )^{2n}|k|^{2n}}{\lambda (k)^{2n}} \;\longrightarrow \; 1 \qquad (|k|\rightarrow \infty ) . \end{aligned}$$

Consequently $\partial ^\alpha u$ is, modulo the field $E_\alpha W$, a variable-order Matérn field with order function m-|α| carried by the bounded multiplier $\theta _\alpha$, and the computation above applies to it with Lemma A.17 used with the weight $w=|\theta _\alpha |^2$. Since $E_\alpha W$ has variogram of strictly smaller order, the Cauchy–Schwarz inequality $\square _h C_{Z_1+Z_2} = \square _h C_{Z_1}\bigl (1+\mathcal {O}(\sqrt{\square _h C_{Z_2}/\square _h C_{Z_1}})\bigr )$ shows that it does not affect either bound. Differentiating *n* times replaces *H* by H-n; choosing $n = \lceil \gamma _V\rceil -1$ brings the reduced exponent into (0, 1]. Induction on *n* now gives (i) and (ii) in general.

Finally, let $\chi \in C_c^\infty (V)$, $\chi \not \equiv 0$. Then $\mathbb {E}\Vert \chi u\Vert _{L^2}^2 = \int \chi (x)^2 C_u(x,x)\,dx = \infty$ by Proposition 4.4, since $C_u(x,x)=+\infty$ on *V*. The event $\{\Vert \chi u\Vert _{L^2}<\infty \}$ is a measurable linear subspace of the sample space, so by the Gaussian zero–one law [14] it has probability 0 or 1; if it had probability 1 then Fernique’s theorem [15] would force $\mathbb {E}\Vert \chi u\Vert _{L^2}^2<\infty$. Hence it has probability 0.

Finally, the concluding identification of the exponent at $x_0$ follows from (i) and (ii) on letting *V* shrink to $x_0$: $\gamma _V \rightarrow H(x_0)$ because *m* is continuous. □

### Locality

#### Theorem 4.6

(Regularity is a local invariant of the order function) Let *A* and $\tilde{A}$ be elliptic pseudo-differential operators on $\mathbb {T}^d$ of variable orders *m* and $\tilde{m}$ respectively, in the sense of Definition 4.1 with some δ<1, and suppose their full symbols agree on an open set $V\subseteq \mathbb {T}^d$. Let $u,\tilde{u}$ be the GeRFs solving $Au {\mathop {=}\limits ^{\text {2nd o.}}}W$ and $\tilde{A}\tilde{u} {\mathop {=}\limits ^{\text {2nd o.}}}W$ for the same white noise *W*. Then $u - \tilde{u}$ has a modification that is almost surely $C^\infty$ on *V*.

Consequently the conclusions of Theorem 4.5 at $x_0$ depend on *m* only through its germ at $x_0$.

#### Proof

Let $$\chi ,\tilde{\chi }\in C_c^\infty (V)$$ with $$\tilde{\chi }\equiv 1$$ on a neighbourhood of $${{\,\textrm{supp}\,}}\chi $$. Write $$B:= A^{-1}-\tilde{A}^{-1}$$, where the inverses are taken modulo smoothing operators: by Lemma A.13 an operator that is elliptic of variable order in the sense of Definition 4.1 admits a parametrix, an operator *P* with PA=I+S and $AP=I+S^{\prime }$ for smoothing $S,S^{\prime }$, whose symbol is built from the full symbol of *A* by the asymptotic inversion (A4); $$A^{-1}$$ denotes such a *P*, and any two choices differ by a smoothing operator. Then

$$ \chi B \;=\; \chi B \tilde{\chi } \;+\; \chi B(1-\tilde{\chi }). $$

The second term is smoothing because pseudo-differential operators in $S_{1,\delta }$ with δ<1 are pseudo-local (their Schwartz kernels are smooth off the diagonal, by the toroidal kernel estimates of [16] (see also Lemma A.13)) and χ and $1-\tilde{\chi }$ have disjoint supports. For the first term, the parametrix symbol (A4) at a point x∈V is determined by the full symbol of *A* at *x* alone; since the full symbols of *A* and $\tilde{A}$ agree on *V*, the two parametrix symbols agree on *V*, and $\chi B\tilde{\chi }$ is smoothing as well. Hence $\chi (u-\tilde{u}) = \chi B W$ is the image of white noise under a smoothing operator, i.e. a Gaussian field with $C^\infty$ covariance kernel, and therefore has an almost surely $C^\infty$ modification. □

## The canonical field: a closed-form check and a rigidity statement

Section 4 determines the exponent H(x)=m(x)-d/2 from a two-sided bound on the variogram. One member of this variable-order family has a covariance that can be written down in closed form: the canonical field, which is the Gaussian free field of the torus. This section computes that covariance, uses it to verify the threshold and the exponent of Sect. 4 by direct calculation, and then proves a rigidity theorem for the fields obtained from it by spectral reweighting.

### The canonical field

On $\mathbb {T}^d$ the operator -Δ annihilates the constants and therefore has no inverse; but it has a Green’s function. Let $G_{-\Delta } \in \mathcal {S}'(\mathbb {T}^d)$ be the unique mean-zero solution of

18 $$\begin{aligned} -\Delta \, G_{-\Delta } \;=\; \delta _0 - 1 , \end{aligned}$$

the constant 1 on the right being the density of the uniform measure, whose subtraction is forced by the compatibility condition $\int _{\mathbb {T}^d}(-\Delta f)=0$.

#### Definition 5.1

*(Canonical field)* The canonical field *G* over $\mathbb {T}^d$ is the centred stationary GeRF with covariance distribution

19 $$\begin{aligned} C_G \;=\; 4\pi ^2\, G_{-\Delta } . \end{aligned}$$

The spectral measure of *G* is read off (18) by expanding in the eigenbasis of -Δ. Since $-\Delta e^{2\pi i k\cdot h} = 4\pi ^2|k|^2\, e^{2\pi i k\cdot h}$ and $\delta _0 - 1 = \sum _{k\ne 0}e^{2\pi i k\cdot h}$ on $\mathbb {T}^d$, equation (18) has the unique mean-zero solution $G_{-\Delta }(h) = \sum _{k\ne 0}(4\pi ^2|k|^2)^{-1}e^{2\pi i k\cdot h}$, whence by (19)

20 $$\begin{aligned} \mu _G(\{k\}) = {\left\{ \begin{array}{ll} |k|^{-2}, &  k \ne 0, \\ 0, &  k = 0 .\end{array}\right. } \end{aligned}$$

Moreover $\mu _G \in \mathcal {M}^+_{SG}(\mathbb {Z}^d)$ in every dimension, so *G* exists as a GeRF for every *d*.

#### Proposition 5.2

(Covariance of the canonical field) The covariance distribution of *G* is $C_G(h) = \sum _{k \in \mathbb {Z}^d\setminus \{0\}} |k|^{-2} e^{2\pi i k\cdot h}$, and

(i) for d=1 and $$h \in [-\tfrac{1}{2},\tfrac{1}{2}]$$, $$\;C_G(h) = \tfrac{\pi ^2}{3} - 2\pi ^2|h| + 2\pi ^2 h^2$$;
(ii) for d=2, $\;C_G(h) = -2\pi \log |h| + O(1)$ as h→0;
(iii) for d≥3, $\;C_G(h) = c_d|h|^{2-d} + o\bigl (|h|^{2-d}\bigr )$ as h→0, with $c_d = \pi ^{2-d/2}\,\Gamma \bigl (\tfrac{d}{2} - 1\bigr )$.

In particular $C_G$ is a bounded continuous function if and only if d=1.

#### Proof

Part (i) is the Fourier series of the second Bernoulli polynomial,

$$ \sum _{k\ge 1} k^{-2}\cos (2\pi k h) \;=\; \pi ^2\bigl (h^2-h+\tfrac{1}{6}\bigr ), \qquad h \in [0,1], $$

of which $C_G(h)$ is twice the sum.

For (ii) and (iii) we compare (18) with its Euclidean counterpart. Let *E* be the Newtonian kernel on $\mathbb {R}^d$, the fundamental solution of -Δ:

$$ E(h) \;=\; -\tfrac{1}{2\pi }\log \Vert h\Vert \quad (d=2), \qquad E(h) \;=\; \frac{\Gamma \bigl (\tfrac{d}{2}-1\bigr )}{4\pi ^{d/2}}\,\Vert h\Vert ^{2-d} \quad (d\ge 3). $$

The torus is flat, so a ball *B* about the origin of radius $<\tfrac{1}{2}$ is isometric to a Euclidean ball and $-\Delta (4\pi ^2 E) = 4\pi ^2\delta _0$ holds in $\mathcal {D}'(B)$. Put $Q(h):= \tfrac{2\pi ^2}{d}\Vert h\Vert ^2$, so that $-\Delta Q = -4\pi ^2$. By (18) and (19),

$$ -\Delta \bigl (C_G - 4\pi ^2E - Q\bigr ) \;=\; 4\pi ^2\delta _0 - 4\pi ^2 - 4\pi ^2\delta _0 + 4\pi ^2 \;=\; 0 \qquad \text {in } \mathcal {D}'(B), $$

so $C_G - 4\pi ^2E - Q$ is a harmonic distribution on *B* and hence, by Weyl’s lemma, a real-analytic function there; in particular it is bounded near 0. Therefore $C_G(h) = 4\pi ^2 E(h) + \mathcal {O}(1)$ as h→0, and multiplying the displayed constants by $4\pi ^2$ gives (ii) and (iii), the error being $\mathcal {O}(1)$ and hence $o(|h|^{2-d})$ when d≥3. Boundedness fails for d≥2 because $C_G(0) = \sum _{k \ne 0}|k|^{-2}$ diverges there. □

The point of the computation is the following check, in which the exponent of Theorem 4.5 is compared with a covariance in closed form.

#### Proposition 5.3

(Closed-form check on the threshold) Take m≡1, so that H≡1-d/2 and the threshold m>d/2 of Proposition 4.4 reads d<2. Then:

(i) for d=1, $\mathbb {E}|G(x)-G(y)|^2 = 4\pi ^2|h| - 4\pi ^2\,h^2$ with h=x-y, so the variogram is of order $|h|^{2\,H}$ with $H = \tfrac{1}{2}$ and the sample-path Hölder exponent is $\tfrac{1}{2}$, as Theorem 4.5 predicts;
(ii) for d=2 the threshold is attained with equality and $C_G(0) = +\infty$, so the field is not a function, consistent with Proposition 4.4;
(iii) for d≥3, m<d/2 everywhere and Theorem 4.5(iii) applies, so $G \notin L^2_{loc}$ almost surely; Proposition 5.2(iii) quantifies the rate at which the diagonal diverges.

#### Proof

Only (i) requires computation: $\mathbb {E}|G(x)-G(y)|^2 = 2\bigl (C_G(0)-C_G(h)\bigr )$, which by Proposition 5.2(i) equals $4\pi ^2|h| - 4\pi ^2h^2 \asymp |h|$ for $|h| \le \tfrac{1}{4}$. Parts (ii) and (iii) are immediate from Proposition 5.2 and the results cited. □

#### Remark 5.4

The exponent $\tfrac{1}{2}$ in (i) identifies the canonical field on $\mathbb {T}^1$ with the Brownian bridge up to normalisation and centring: the covariance in Proposition 5.2(i) is $4\pi ^2\bigl (\tfrac{1}{12} - \tfrac{1}{2}|h| + \tfrac{1}{2}h^2\bigr )$.

### Rational reweighting of the spectral density

Applying the canonical factor $|k|^{-2}$ to the Matérn spectral density produces a field of higher smoothness. Theorem 5.6 identifies that field. It is an absolutely convergent superposition of ordinary Matérn fields whose leading term has smoothness ν+1. This shows that the Matérn class is closed under reweighting by the reciprocal of a polynomial in $|k|^2$ with nonnegative coefficients, and that the resulting shift in smoothness is the degree of that polynomial.

#### Definition 5.5

*(Canonical-Matérn field)* For α>0 the canonical-Matérn field $u^{(\alpha )}$ is the stationary solution of $\mathcal {L}^{\alpha /2}u^{(\alpha )} {\mathop {=}\limits ^{\text {2nd o.}}}G$, equivalently the stationary GeRF with spectral measure

21 $$\begin{aligned} \mu ^{(\alpha )}_{CM}(\{k\}) \;=\; |k|^{-2}\lambda (k)^{-2\alpha } \quad (k \ne 0), \qquad \mu ^{(\alpha )}_{CM}(\{0\}) = 0 . \end{aligned}$$

#### Theorem 5.6

(Rigidity of the canonical factor) For every $\alpha \in \mathbb {R}$ and every k≠0 the identity (22) below holds between the measures (21); the statements about the field $u^{(\alpha )}$ are for α>0. Precisely,

22 $$\begin{aligned} \mu ^{(\alpha )}_{CM}(\{k\}) \;=\; 4\pi ^2\,\lambda (k)^{-2(\alpha +1)} \;+\; \kappa ^2\,\mu ^{(\alpha +1)}_{CM}(\{k\}) , \end{aligned}$$

and iterating gives the absolutely convergent expansion

23 $$\begin{aligned} \mu ^{(\alpha )}_{CM}(\{k\}) \;=\; 4\pi ^2 \sum _{j \ge 0} \kappa ^{2j}\, \lambda (k)^{-2(\alpha +1+j)} , \qquad k \ne 0 . \end{aligned}$$

Consequently, writing $X^{\perp }_{\nu }$ for the Matérn field of smoothness ν with the constant mode removed and $\nu = \alpha - d/2$,

24 $$\begin{aligned} u^{(\alpha )} \;{\mathop {=}\limits ^{\text {2nd o.}}}\; 2\pi \sum _{j\ge 0} \kappa ^{j}\,X^{\perp }_{\nu +1+j} , \end{aligned}$$

the summands being independent and the series converging in $L^2(\Omega )$ after testing against any $\varphi \in \mathcal {S}(\mathbb {T}^d)$. In particular $u^{(\alpha )} {\mathop {=}\limits ^{\text {2nd o.}}}2\pi X^{\perp }_{\nu +1} + R$ with *R* independent of $X^\perp _{\nu +1}$ and of smoothness ν+2, one unit of ν smoother; and if ν+1>0 then $u^{(\alpha )}$ has samples in $C^{(\nu +1)^-}_{loc}$ and no more.

#### Proof

For k≠0 we have $\lambda (k)^2 - \kappa ^2 = 4\pi ^2|k|^2 > 0$, so

$$ \frac{1}{|k|^2} \;=\; \frac{4\pi ^2}{\lambda (k)^2 - \kappa ^2} \;=\; \frac{4\pi ^2}{\lambda (k)^2} + \frac{\kappa ^2}{|k|^2\lambda (k)^2}, $$

and multiplying by $\lambda (k)^{-2\alpha }$ gives (22). Iterating and summing the geometric series in $\kappa ^2/\lambda (k)^2 \le \kappa ^2/(\kappa ^2+4\pi ^2) < 1$ gives (23), the convergence being uniform in k≠0. Every term of (23) is nonnegative, so the expansion exhibits $\mu ^{(\alpha )}_{CM}$ as a sum of spectral measures; a sum of spectral measures is the spectral measure of a sum of independent stationary GeRFs, and the *j*-th summand, $4\pi ^2\kappa ^{2j}\lambda ^{-2(\alpha +1+j)}$ off the origin, is the spectral measure of $2\pi \kappa ^{j}X^{\perp }_{\nu +1+j}$. This is (24). The regularity statement follows from Lemma A.16 with $s = \nu + \tfrac{d}{2} + 1$, since (21) gives $\mu ^{(\alpha )}_{CM} \asymp \lambda ^{-2s}$ away from the origin; sharpness follows from that of Proposition 3.4 applied to the leading summand, the remainder being smoother by one unit of ν and therefore unable to affect the exponent. □

## Conclusion

This paper has examined how the properties of Matérn covariance functions in Euclidean space transfer to manifold settings, focusing on the tractable case of tori, and has then used the same machinery to construct new fields. Through the framework of pseudo-differential operators on tori, we have established both theoretical results and practical consequences for spatial modelling on closed manifolds.

Three things have been established. The first is that the stationary theory holds no surprises: a Matérn process on $\mathbb {T}^d$ with smoothness parameter ν>0 has sample paths in $C^{\nu ^-}_{loc}$ and no more, the same exponent as in $\mathbb {R}^d$. The pseudo-differential proof is nonetheless worth giving, because it uses only the order of the symbol and therefore could be useful in settings where no closed form is available.

The second, and the substance of the paper, is that regularity on the torus becomes local as soon as the order of the symbol is allowed to vary with position. For an order function *m*, the field $\textrm{Op}(\lambda ^{-m(\cdot )})W$ has pointwise Hölder exponent m(x)-d/2, exists as a function exactly where m(x)>d/2, and has a regularity near any point that depends on *m* only through its germ there. The proof rests on the increment criterion of Theorem 3.2 and the symbol calculus, and should carry to more general compact manifolds by charts and a partition of unity, since the variable-order symbol class is coordinate-invariant modulo lower-order terms.

The third result concerns spectral reweighting. Weighting the Matérn spectral density by $|k|^{-2}$, the symbol of the inverse Laplacian, produces a field of regularity $C^{(\nu +1)^-}_{loc}$. The field is an explicit convergent superposition of ordinary Matérn fields of smoothnesses $\nu +1,\nu +2,\dots$, its leading term a Matérn field of smoothness ν+1 with the constant mode removed and the remainder smoother by one further unit of ν.

The practical implications are as follows. For stationary models on periodic domains, Euclidean intuition about the smoothness parameter transfers intact. What the variable-order construction adds is a model class in which smoothness itself is a free function of position. Since the threshold is local, such a model may be specified aggressively in one region without losing regularity guarantees elsewhere. Global constructions, whose guarantees are governed by the least smooth point of the domain, cannot have this property.

Two directions for future research emerge naturally from this work. First, the results of Sect. 4 are stated on the torus, where the quantization is cleanest, but the variable-order symbol class is coordinate-invariant modulo lower-order terms; carrying Theorems 4.5 and 4.6 to a general closed manifold by charts and a partition of unity is the natural next step. Second, the inference problem is open. Estimating an order function from a single realisation is a nonparametric problem with no established theory.

Finally, this work demonstrates the value of rigorous mathematical analysis in spatial statistics. While computational tools like INLA and the SPDE approach have greatly expanded the practical applicability of Matérn models, understanding the theoretical properties of these models on non-Euclidean domains remains essential for their appropriate use. We hope this paper provides both theoretical foundations for future work on spatial modeling on manifolds and practical guidance for applied researchers working with spatial data on periodic and compact domains.

## Data Availability

No datasets were generated or analysed during the current study.

## Abstract theory for GeRFs on tori

This appendix collects the abstract functional-analytic foundations that support the main results. It also provides the complete theoretical framework for Generalized Random Fields on tori.

### Kernel representation theory

The functional analytic foundations for GeRF theory benefit from Ehrenpreis’s kernel representation theorem, which extends naturally to the torus setting with significant simplifications.

#### Lemma A.1

(Decomposition Lemma for Tori) Let *B* be a bounded set in $\mathcal {S}(\mathbb {T}^d \times \mathbb {T}^d)$. Then we can find a bounded set $B' \subset \mathcal {S}(\mathbb {T}^d)$ and a constant b>0 such that every f∈B can be written as:

$$\begin{aligned} f = \sum _{j_1,j_2} \lambda _{j_1,j_2} g_{j_1} \times h_{j_2} \end{aligned}$$

where $\sum _{j_1,j_2} |\lambda _{j_1,j_2}| \le b$, and $g_{j_1}, h_{j_2} \in B'$, with the series converging in $\mathcal {S}(\mathbb {T}^d \times \mathbb {T}^d)$.

#### Proof Sketch

For functions on tori, the Fourier series expansion is immediate:

$$\begin{aligned} f(x,y) = \sum _{j_1,j_2 \in \mathbb {Z}^d} c_{j_1,j_2} e^{2\pi i j_1 \cdot x} e^{2\pi i j_2 \cdot y} \end{aligned}$$

The rapid decrease of Fourier coefficients for smooth functions ensures uniform bounds. The decomposition is achieved by setting:

A1 $$\begin{aligned} g_{j_1}(x)&= |c_{j_1,j_2}|^{1/3} e^{2\pi i j_1 \cdot x} \end{aligned}$$

A2 $$\begin{aligned} h_{j_2}(y)&= |c_{j_1,j_2}|^{1/3} e^{2\pi i j_2 \cdot y} \end{aligned}$$

A3 $$\begin{aligned} \lambda _{j_1,j_2}&= |c_{j_1,j_2}|^{1/3} \end{aligned}$$

□

#### Theorem A.2

(Kernel Representation on Tori) Let $\mathscr {J}_{\mathbb {T}}$ be the space of continuous linear maps $\mathcal {S}(\mathbb {T}^d) \rightarrow \mathcal {S}'(\mathbb {T}^d)$ with the compact-open topology. Then the map $t: \mathcal {S}'(\mathbb {T}^d \times \mathbb {T}^d) \rightarrow \mathscr {J}_{\mathbb {T}}$ which assigns to each distribution *S* the linear map *L* such that *S* is a kernel representing *L* is a topological isomorphism onto.

#### Proof

Same as in [17]. The key point in that proof that needed adaptation to the Torus setting was the lemma presented above. □

### Bochner–Schwartz theory for Abelian groups

The theoretical foundations are further strengthened by Wawrzyńczyk’s extension of the Bochner–Schwartz theorem to arbitrary locally compact Abelian groups [18], which provides crucial insights for the torus case.

#### Theorem A.3

(Wawrzyńczyk’s Extension Theorem, [18]) For a separable locally compact Abelian group *G* with character group Γ:

1. Every positive-definite tempered distribution *T* on *G* can be represented by a positive Borel measure *m* on Γ:
  $$\begin{aligned} T(\varphi ) = \int _\Gamma \hat{\varphi }(\gamma ) \, dm(\gamma ) \end{aligned}$$
2. The measure *m* can be extended uniquely to a tempered distribution on Γ.

For the torus $\mathbb {T}^d$, this implies:

#### Corollary A.4

(Discrete Extension Property) For the torus, the measure μ on $\mathbb {Z}^d$ extends to a tempered distribution naturally via:

$$\begin{aligned} \tilde{T}(\psi ) = \sum _{k \in \mathbb {Z}^d} \psi (k) \mu (\{k\}) \end{aligned}$$

for any rapidly decreasing sequence $\psi \in \mathcal {S}(\mathbb {Z}^d)$.

### Detailed GeRF theory

We now develop the complete theory of Generalized Random Fields on tori, including characterizations via orthogonal random measures and operator actions.

The class $\mathcal {M}_{SG}^+(\mathbb {Z}^d)$ of slowly growing measures was defined in (2); the results below identify it as exactly the class of spectral measures of stationary GeRFs.

Let *Z* be a GeRF on $\mathbb {T}^d$. As a consequence of Theorem A.2, there are distributions $m_Z$ and $C_Z$ such that $\mathbb {E}[\langle Z, \varphi \rangle ] = \langle m_Z, \varphi \rangle$ and

$$\begin{aligned} \mathbb {C}\textrm{ov}(\langle Z, \varphi \rangle , \langle Z, \phi \rangle ) = \langle C_Z, \varphi \otimes \bar{\phi } \rangle . \end{aligned}$$

In what follows, we assume $m_Z = 0$.

The spectral measure exists by Theorem A.3.

#### Definition A.5

*(Orthogonal Random Measure)* A collection of random variables $\{W(k)\}_{k \in \mathbb {Z}^d}$ is an orthogonal random measure on $\mathbb {Z}^d$ with weight $\mu \in \mathcal {M}_{SG}^+(\mathbb {Z}^d)$ if:

$$\begin{aligned} \mathbb {C}\textrm{ov}(W(j), W(k)) = \delta _{j,k} \mu (\{j\}) \end{aligned}$$

where $\delta _{j,k}$ is the Kronecker delta. In addition, an orthogonal random measure $\{W(k)\}_{k \in \mathbb {Z}^d}$ is slow-growing if there exist constants C>0 and $N \in \mathbb {N}$ such that:

$$\begin{aligned} \mathbb {E}[|W(k)|^2] \le C(1 + |k|^2)^N \quad \text {for all } k \in \mathbb {Z}^d \end{aligned}$$

The following results provide complete characterizations of stationary GeRFs.

#### Proposition A.6

(Fourier transform characterization) Let *Z* be a real stationary GeRF over $\mathbb {T}^d$ with spectral measure $\mu _Z \in \mathcal {M}_{SG}^+(\mathbb {Z}^d)$. Then the Fourier coefficients $\{\hat{Z}(k)\}_{k \in \mathbb {Z}^d}$ defined by

$$\begin{aligned} \hat{Z}(k) = \langle Z, e^{-2\pi i k \cdot } \rangle \end{aligned}$$

form a Hermitian-symmetric complex slow-growing orthogonal random measure on $\mathbb {Z}^d$ with weight $\mu _Z$.

#### Proof

Write $e_k(x):= e^{-2\pi i k\cdot x}$. Since $\overline{e_{k'}} = e_{-k'}$ and $\check{\overline{e_{k'}}} = e_{k'}$, the convolution

$$ \bigl (e_k *\check{\overline{e_{k'}}}\bigr )(x) \;=\; \int _{\mathbb {T}^d} e^{-2\pi i k\cdot (x-y)}\,e^{-2\pi i k'\cdot y}\,dy \;=\; e_k(x)\int _{\mathbb {T}^d} e^{2\pi i (k-k')\cdot y}\,dy \;=\; \delta _{k,k'}\,e_k(x), $$

the last step by orthogonality of the characters on $\mathbb {T}^d$, which carries total mass 1. Hence, by (6),

$$ \mathbb {C}\textrm{ov}\bigl (\hat{Z}(k),\hat{Z}(k')\bigr ) \;=\; \bigl \langle C_Z, e_k \otimes \overline{e_{k'}}\bigr \rangle \;=\; \bigl \langle \rho _Z, e_k *\check{\overline{e_{k'}}}\bigr \rangle \;=\; \delta _{k,k'}\,\langle \rho _Z, e_k\rangle \;=\; \delta _{k,k'}\,\hat{\rho }_Z(k), $$

and $\hat{\rho }_Z(k) = \mu _Z(\{k\})$ because $\rho _Z = \mathscr {F}^{-1}(\mu _Z)$. This is the orthogonality relation with weight $\mu _Z$. Hermitian symmetry follows from *Z* being real: $\overline{\hat{Z}(k)} = \langle Z, \overline{e_k}\rangle = \langle Z, e_{-k}\rangle = \hat{Z}(-k)$. Finally $\mathbb {E}|\hat{Z}(k)|^2 = \mu _Z(\{k\})$, and (2) forces $\mu _Z(\{k\}) \le C\langle k\rangle ^{2N}$ for some *C*, *N*, which is the slow-growth condition. □

#### Proposition A.7

(Operator action) Let *U* be a real stationary GeRF on $\mathbb {T}^d$ with spectral measure $\mu _U$ and let *g* be a symbol function. Then $\textrm{Op}(g) U$ is a real stationary GeRF with spectral measure $|g|^2 \mu _U$ and covariance distribution $\rho _{\textrm{Op}(g) U} = \textrm{Op}(|g|^2)\rho _U$.

#### Proof

By definition $\langle \textrm{Op}(g)U,\varphi \rangle = \langle U, \textrm{Op}(\check{g})\varphi \rangle$, and $\widehat{\textrm{Op}(\check{g})\varphi }(k) = \check{g}(k)\hat{\varphi }(k)$. Expanding $\varphi = \sum _k \hat{\varphi }(k)\,e^{2\pi i k\cdot }$ and using Proposition A.6 together with the continuity of *U*,

$$ \mathbb {C}\textrm{ov}\bigl (\langle \textrm{Op}(g)U,\varphi \rangle ,\langle \textrm{Op}(g)U,\phi \rangle \bigr ) \;=\; \sum _{k\in \mathbb {Z}^d} |\check{g}(k)|^2\,\hat{\varphi }(k)\, \overline{\hat{\phi }(k)}\;\mu _U(\{k\}), $$

the series converging absolutely because *g* has polynomial growth and $\hat{\varphi },\hat{\phi }$ decrease rapidly. Since $\textrm{Op}(g)$ preserves real distributions we have $\check{g} = \bar{g}$, so $|\check{g}| = |g|$, and the displayed formula is (6) for the stationary field with spectral measure $|g|^2\mu _U$. That measure lies in $\mathcal {M}^+_{SG}(\mathbb {Z}^d)$ because $|g|^2$ is of polynomial growth and $\mu _U$ satisfies (2). Taking inverse Fourier transforms gives $\rho _{\textrm{Op}(g)U} = \mathscr {F}^{-1}(|g|^2\mu _U) = \textrm{Op}(|g|^2)\rho _U$. □

#### Theorem A.8

(Existence and Uniqueness) Let *X* be a real stationary GeRF on $\mathbb {T}^d$ with spectral measure $\mu _X$. Let *g* be a symbol function and $\textrm{Op}(g)$ the associated operator. Then:

(i) There exists a real stationary GeRF *U* satisfying $\textrm{Op}(g) U {\mathop {=}\limits ^{\text {2nd o.}}}X$ if and only if there exists $N \in \mathbb {N}$ such that
  $$\begin{aligned} \sum _{k \in \mathbb {Z}^d} \frac{\mu _X(\{k\})}{|g(k)|^2(1+|k|^2)^N} < \infty . \end{aligned}$$
(ii) When existence holds, the measure
  $$\begin{aligned} \mu _U(\{k\}) = \frac{\mu _X(\{k\})}{|g(k)|^2} \end{aligned}$$
  (with the convention that $\mu _U(\{k\}) = 0$ when g(k)=0) belongs to $\mathcal {M}_{SG}^+(\mathbb {Z}^d)$ and is even. Any real stationary GeRF with spectral measure $\mu _U$ solves $\textrm{Op}(g) U {\mathop {=}\limits ^{\text {2nd o.}}}X$.
(iii) The spectral measure $\mu _U$ is unique in $\mathcal {M}_{SG}^+(\mathbb {Z}^d)$ if and only if |g(k)|>0 for all $k \in \mathbb {Z}^d$.

#### Proof

The proof follows the same structure as in [11], with straightforward adaptations to the discrete setting of the torus. □

#### Remark A.9

The condition |g(k)|>0 for all $k \in \mathbb {Z}^d$ is equivalent to the invertibility of the operator $\textrm{Op}(g)$. In this case, the unique solution is given explicitly by $U = \textrm{Op}(1/g)X$.

White Noise on the torus serves as a fundamental building block for more general solutions.

#### Definition A.10

*(White Noise on Tori)* The White Noise *W* on $\mathbb {T}^d$ is the real GeRF characterized by the covariance:

$$\begin{aligned} \langle C_W, \varphi \otimes \overline{\phi } \rangle = \int _{\mathbb {T}^d} \varphi (x)\overline{\phi (x)} dx \end{aligned}$$

for all $$\varphi , \phi \in \mathcal {S}(\mathbb {T}^d)$$.

This implies that *W* is stationary with covariance distribution $\rho _W(x) = \delta _0(x)$, where $\delta _0$ is the point mass at zero. Hence:

#### Proposition A.11

(Spectral measure of white noise) The spectral measure of White Noise on $\mathbb {T}^d$ assigns weight $\mu _W(\{k\}) = 1$ to each frequency $k \in \mathbb {Z}^d$.

#### Proof

By Sect. 2, $$\mu _W(\{k\}) = \hat{\rho }_W(k) = \langle \delta _0, e^{-2\pi i k\cdot }\rangle = 1$$. □

The following result describes how covariance functions behave under operator transformations, showing that they have a natural convolution structure.

#### Theorem A.12

(Convolution structure on tori) Let *X* be a real stationary GeRF on $\mathbb {T}^d$ with covariance distribution $\rho _X$. Let *g* be a symbol function on $\mathbb {Z}^d$ such that the sequences $\{1/g(k)\}_{k \in \mathbb {Z}^d}$ and $\{|g(k)|^{-2}\}_{k \in \mathbb {Z}^d}$ have polynomial growth. Then there exists a unique stationary solution to

$$\begin{aligned} \textrm{Op}(g) U {\mathop {=}\limits ^{\text {2nd o.}}}X \end{aligned}$$

and its covariance distribution is given by:

$$\begin{aligned} \rho _U = \rho _U^W * \rho _X \end{aligned}$$

where $\rho _U^W$ is the covariance of the unique stationary solution to $\textrm{Op}(g) U {\mathop {=}\limits ^{\text {2nd o.}}}W$.

#### Proof

The proof follows the same approach as in [11]. □

This convolution structure provides an alternative perspective on how solutions to operator equations inherit regularity from white noise.

### The variable-order calculus: parametrix and pseudo-locality

The locality theorem, Theorem 4.6, rests on two facts about the class of Definition 4.1. Both are the toroidal statements of [12, 13] and [16]; we record them in the variable-order form in which they are used.

#### Lemma A.13

(Parametrix and pseudo-locality) Let $0 \le \delta < 1$, let $m \in C^\infty (\mathbb {T}^d;\mathbb {R})$ and let $p \in S^{m(\cdot )}_{1,\delta }(\mathbb {T}^d\times \mathbb {Z}^d)$ be elliptic of variable order *m* in the sense of Definition 4.1. Then:

(i) The Schwartz kernel of $\textrm{Op}(p)$ is $C^\infty$ off the diagonal of $\mathbb {T}^d\times \mathbb {T}^d$.
(ii) There is $$q \in S^{-m(\cdot )}_{1,\delta }(\mathbb {T}^d\times \mathbb {Z}^d)$$ with $$\textrm{Op}(q)\textrm{Op}(p) = I + S$$ and $$\textrm{Op}(p)\textrm{Op}(q) = I + S'$$, where $S,S^{\prime }$ are smoothing. The symbol *q* admits the asymptotic expansion $$q \sim \sum _{N\ge 0}q_N$$ determined by
  A4 $$\begin{aligned} q_0(x,k) = \frac{\chi (k)}{p(x,k)}, \qquad q_N(x,k) = -\,q_0(x,k)\!\!\sum _{0<|\alpha |\le N}\frac{1}{\alpha !}\, \Delta _k^{\alpha }p(x,k)\; D_x^{\alpha } q_{N-|\alpha |}(x,k) , \end{aligned}$$
  where χ vanishes for $|k| < k_0$ and equals 1 for $|k| \ge k_0+1$, and $D_x^{\alpha }$ is the differential operator in *x* occurring in the toroidal composition formula of [12, 13]. One has $q_N \in S^{-m(\cdot )-N(1-\delta )}_{1,\delta }$.

In particular, the parametrix symbol at a point *x* is determined by the full symbol of *p* at *x* alone.

#### Proof

Part (i) is the toroidal kernel estimate of [16]: for $p \in S^{a}_{1,\delta }$ with δ<1 and any *N*, repeated summation by parts in *k* against the factor $(e^{2\pi i(x-y)\cdot k}-1)^{-1}$ converts decay in |*k*| into smoothness of the kernel away from x=y; since *m* is continuous on the compact torus it is bounded, so $S^{m(\cdot )}_{1,\delta } \subseteq S^{a}_{1,\delta }$ with $a = \max _x m(x)$ and the cited estimate applies.

For (ii), ellipticity makes $q_0$ well defined and gives $q_0 \in S^{-m(\cdot )}_{1,\delta }$: the bound $|q_0| \le c^{-1}\langle k\rangle ^{-m(x)}$ is the ellipticity hypothesis, and the estimates on $\Delta _k^{\alpha }\partial _x^{\beta }q_0$ follow from those on *p* by the quotient rule together with the Leibniz rules for $\Delta _k$ and $\partial _x$. The class is an algebra in the sense that $S^{a(\cdot )}_{1,\delta }\cdot S^{b(\cdot )}_{1,\delta } \subseteq S^{a(\cdot )+b(\cdot )}_{1,\delta }$, again by the Leibniz rules, so an induction on *N* using (A4) gives $q_N \in S^{-m(\cdot )-N(1-\delta )}_{1,\delta }$. Because $\sup _x m(x) < \infty$, the orders -m(x)-N(1-δ) tend to -∞ uniformly in *x*, so the asymptotic sum may be realised by the usual Borel-type summation, and the composition formula of [12, 13] then gives $\textrm{Op}(q)\textrm{Op}(p)-I$ and $\textrm{Op}(p)\textrm{Op}(q)-I$ of order -∞. Every step of (A4) evaluates *p* and its derivatives at the same base point *x*, which is the last assertion. □

### Technical lemmas

This subsection collects the estimates the main body relies on. The first two concern the Matérn kernel: its exponential decay, and its expansion near the origin. We write ℓ>0 for the range parameter and $\kappa = \sqrt{2\nu }/\ell$ as in (1).

#### Lemma A.14

(Exponential decay of matérn kernel) The Euclidean Matérn kernel with smoothness parameter ν>0 and range parameter ℓ>0 is

$$\begin{aligned} k^{\mathbb {R}}_\nu (r) = \sigma ^2 \frac{2^{1-\nu }}{\Gamma (\nu )} \left( \frac{r\sqrt{2\nu }}{\ell }\right) ^\nu K_\nu \left( \frac{r\sqrt{2\nu }}{\ell }\right) , \end{aligned}$$

where $K_\nu$ is the modified Bessel function of the second kind. For every $j \in \mathbb {N}_0$ there is $C_j = C_j(\nu ,\ell ,\sigma )$ with

$$\Bigl |\tfrac{d^j}{dr^j}k^{\mathbb {R}}_\nu (r)\Bigr | \le C_j \, r^{\nu -1/2} \, e^{-\kappa r} \qquad \text {for } r \ge 1.$$

#### Proof

Write z=κr with $\kappa = \sqrt{2\nu }/\ell$, so that $k^{\mathbb {R}}_\nu (r) = c_{\nu ,\sigma }\, z^\nu K_\nu (z)$. The function $z \mapsto z^\nu K_\nu (z)$ and each of its derivatives satisfy $\bigl |\tfrac{d^j}{dz^j}\bigl (z^\nu K_\nu (z)\bigr )\bigr | \le C_j\, z^{\nu -1/2}e^{-z}$ for $z \ge \kappa$: this is the asymptotic expansion $K_\mu (z) = \sqrt{\pi /2z}\,e^{-z}\bigl (1+O(1/z)\bigr )$ [19], valid for each fixed order μ and differentiable term by term, together with the recurrence $\tfrac{d}{dz}\bigl (z^\mu K_\mu (z)\bigr ) = -z^{\mu }K_{\mu -1}(z)$, which shows that every derivative is again of the same form with μ decreased by one. Undoing the substitution multiplies the *j*-th derivative by $\kappa ^{j}$ and absorbs it into $C_j$. □

#### Lemma A.15

(Local expansion of the matérn kernel) Let ν>0. There are entire functions *A* and *B* with B(0)=1, and a constant $c_\nu \ne 0$, such that for *h* in a neighbourhood of the origin in $\mathbb {R}^d$,

$$ k^{\mathbb {R}}_\nu (h) \;=\; A\bigl (\Vert h\Vert ^2\bigr ) \;+\; c_\nu \,\Vert h\Vert ^{2\nu }\,B\bigl (\Vert h\Vert ^2\bigr ) \cdot {\left\{ \begin{array}{ll} 1, &  \nu \notin \mathbb {N}, \\ \log \Vert h\Vert , &  \nu \in \mathbb {N}. \end{array}\right. } $$

#### Proof

Write $z = \kappa \Vert h\Vert$, so $k^{\mathbb {R}}_\nu (h) = c_{\nu ,\sigma }\,z^{\nu }K_\nu (z)$, and recall the ascending series [19, Eq. 10.25.2]

$$ I_\mu (z) \;=\; \Bigl (\tfrac{z}{2}\Bigr )^{\mu } \sum _{j\ge 0}\frac{(z^2/4)^{j}}{j!\,\Gamma (\mu +j+1)}. $$

If $\nu \notin \mathbb {N}$ then $\nu \notin \mathbb {Z}$ and [19, Eq. 10.27.4] gives $K_\nu = \tfrac{\pi }{2}\bigl (I_{-\nu }-I_\nu \bigr )/\sin (\nu \pi )$. Multiplying by $z^{\nu }$,

$$ z^{\nu }I_{-\nu }(z) = 2^{\nu }\sum _{j\ge 0} \frac{(z^2/4)^{j}}{j!\,\Gamma (-\nu +j+1)}, \qquad z^{\nu }I_{\nu }(z) = 2^{-\nu }z^{2\nu }\sum _{j\ge 0} \frac{(z^2/4)^{j}}{j!\,\Gamma (\nu +j+1)}; $$

the first is an entire function of $z^2$, and the second is $z^{2\nu }$ times an entire function of $z^2$ whose value at the origin is $2^{-\nu }/\Gamma (\nu +1) \ne 0$. Substituting $z^2 = \kappa ^2\Vert h\Vert ^2$, collecting the term without the factor $z^{2\nu }$ into *A*, and absorbing the constants $\kappa ^{2\nu }$, $c_{\nu ,\sigma }$, $-\pi /(2\sin \nu \pi )$ and $2^{-\nu }/\Gamma (\nu +1)$ into $c_\nu$ gives the first case; *B* is then the entire function normalised by B(0)=1, and $c_\nu \ne 0$ because $\sin (\nu \pi )\ne 0$.

If $\nu = n \in \mathbb {N}$, the power series [19, Eq. 10.31.1] reads

$$\begin{aligned} K_n(z) \;=\;&\tfrac{1}{2}\Bigl (\tfrac{z}{2}\Bigr )^{-n} \sum _{j=0}^{n-1}\frac{(n-j-1)!}{j!}\Bigl (-\tfrac{z^2}{4}\Bigr )^{j} \;-\;(-1)^{n}\log \Bigl (\tfrac{z}{2}\Bigr ) I_n(z) \\&\;+\;(-1)^{n}\tfrac{1}{2}\Bigl (\tfrac{z}{2}\Bigr )^{n}\!\! \sum _{j\ge 0}\bigl (\psi (j{+}1)+\psi (n{+}j{+}1)\bigr ) \frac{(z^2/4)^{j}}{j!\,(n+j)!} . \end{aligned}$$

Multiplying by $z^{n}$: the first sum becomes a polynomial in $z^2$; the third becomes $z^{2n}$ times an entire function of $z^2$; and $z^{n}I_n(z) = 2^{-n}z^{2n}\sum _{j\ge 0}(z^2/4)^{j}/\bigl (j!\,(n+j)!\bigr )$, so the middle term is $-(-1)^{n}2^{-n}\bigl (\log z - \log 2\bigr )z^{2n}$ times an entire function of $z^2$ with value 1/*n*! at the origin. Writing $\log z = \log \Vert h\Vert + \log \kappa$ and collecting all terms without a logarithm into $A(\Vert h\Vert ^2)$ gives the second case, with $c_\nu = -(-1)^{n}2^{-n}\kappa ^{2n}c_{\nu ,\sigma }/n! \ne 0$. □

The next estimate converts spectral decay into sample-path regularity.

#### Lemma A.16

(Increment bound for stationary covariances) Let *Z* be a real stationary GeRF on $\mathbb {T}^d$ whose spectral measure satisfies $\mu _Z(\{k\}) \le C_0\,\lambda (k)^{-2s}$ for all $|k| \ge k_0$, where s>d/2. Put $j:= \lceil s - d/2\rceil - 1$, so that r:=2(s-d/2)-2j∈(0,2]. Then $\rho _Z \in C^{2j}(\mathbb {T}^d)$, and for every multi-index α with |α|=2j and every ϵ<r/2,

$$ \bigl |\partial ^\alpha \rho _Z(h) - \partial ^\alpha \rho _Z(0)\bigr | \;=\; \mathcal {O}\bigl (\Vert h\Vert ^{2\epsilon }\bigr ), \qquad h\rightarrow 0. $$

Consequently, by the “if” half of Theorem 3.2, *Z* has samples in $C^{(s-d/2)^-}_{loc}(\mathcal {O})$.

#### Proof

Only the tail matters. The finitely many frequencies $|k| < k_0$ contribute to $\rho _Z$ a trigonometric polynomial, which is smooth and even, so each of its even-order derivatives has vanishing gradient at the origin and its increment there is $\mathcal {O}(\Vert h\Vert ^2)$; since $2\epsilon < r \le 2$ this is absorbed into the claim. We may therefore assume $\mu _Z(\{k\}) \le C_0\lambda (k)^{-2s}$ for all *k*.

Write $\rho _Z(h) = \sum _{k} \mu _Z(\{k\})\, e^{2\pi i k\cdot h}$. Because |α|=2j and 2j-2s+d<0, the differentiated series

A5 $$\begin{aligned} \partial ^\alpha \rho _Z(h) \;=\; \sum _{k} (2\pi i k)^{\alpha }\, \mu _Z(\{k\})\, e^{2\pi i k\cdot h} \end{aligned}$$

converges absolutely and uniformly, whence $\rho _Z \in C^{2j}$. Since *Z* is real and stationary, $\mu _Z$ is even; pairing *k* with -k in (A5) and using that |α| is even replaces the exponential by a cosine, so

$$ \partial ^\alpha \rho _Z(h) - \partial ^\alpha \rho _Z(0) \;=\; \sum _{k} (2\pi i k)^{\alpha }\, \mu _Z(\{k\}) \bigl (\cos (2\pi k\cdot h) - 1\bigr ). $$

For $\theta \in [0,2]$, interpolating $|\cos t - 1| \le 2$ against $|\cos t - 1| \le \tfrac{1}{2} t^2$ gives $|\cos t - 1| \le 2^{1-\theta }|t|^{\theta }$, so $|\cos (2\pi k\cdot h) - 1| \le 2^{1-\theta }(2\pi )^{\theta }|k|^{\theta }\Vert h\Vert ^{\theta }$ and

$$ \bigl |\partial ^\alpha \rho _Z(h) - \partial ^\alpha \rho _Z(0)\bigr | \;\le \; C_\theta \, \Vert h\Vert ^{\theta } \sum _{k} \langle k\rangle ^{\,2j - 2\,s + \theta }. $$

The series converges precisely when 2j-2s+θ+d<0, that is when θ<2(s-d/2)-2j=r. Taking $\theta = 2\epsilon$ gives the display, and j+ϵ ranges up to j+r/2=s-d/2. □

The last estimate is the two-sided lattice bound used in the proof of Theorem 4.5. It is stated with a weight because the induction on the order of differentiation there produces the multipliers (16).

#### Lemma A.17

(Weighted lattice estimate) Let $w: \mathbb {Z}^d \rightarrow [0,\infty )$ satisfy $w \le C_1$ everywhere and $w(k) \ge c_1 > 0$ for $|k| \ge k_1$, and write

$$ S_s(h) \;:=\; \sum _{k\in \mathbb {Z}^d} w(k)\,\lambda (k)^{-2\,s} \bigl (1-\cos 2\pi k\cdot h\bigr ). $$

- If d<2s<d+2 then there are constants $0<c\le C<\infty$ with
  $$ c\,|h|^{2\,s-d} \;\le \; S_s(h) \;\le \; C\,|h|^{2\,s-d} \qquad \text {for } 0 < |h| \le \tfrac{1}{4}. $$
  The constants depend only on $d,c_1,C_1,k_1,\kappa$ and on *s*, and may be chosen uniformly for *s* ranging in a compact subinterval of $\bigl (\tfrac{d}{2},\tfrac{d+2}{2}\bigr )$.
- If 2s=d+2 then $S_s(h) \le C\,|h|^{2}\log (2/|h|)$ for $0<|h|\le \tfrac{1}{4}$. In particular $S_s(h) = \mathcal {O}(|h|^{2\epsilon })$ for every ϵ<1.

For w≡1, part (a) is the periodic form of the classical Bessel potential asymptotics; see [20].

#### Proof

Throughout $\lambda (k) \asymp \langle k\rangle$ with constants independent of *s* once *s* is confined to a bounded set, so it suffices to prove the bounds with $\langle k\rangle ^{-2s}$ in place of $\lambda (k)^{-2s}$. Write *S*(*h*) for $S_s(h)$.

*Upper bound, case *(a)*.* Using $w \le C_1$, split at |k|≤1/|h|. For the near frequencies, $1-\cos t \le t^2/2$ gives

$$ \sum _{|k|\le 1/|h|}\!\!\langle k\rangle ^{-2\,s}\bigl (1-\cos 2\pi k\cdot h\bigr ) \;\le \; 2\pi ^2|h|^2\!\!\sum _{|k|\le 1/|h|}\!\!\langle k\rangle ^{2-2\,s} \;\le \; C|h|^2\,|h|^{\,2\,s-d-2} \;=\; C|h|^{2\,s-d}, $$

where $\sum _{|k|\le R}\langle k\rangle ^{2-2s} \le CR^{\,d+2-2s}$ because d+2-2s>0. For the far frequencies, $1-\cos \le 2$ gives $\sum _{|k|> 1/|h|}\langle k\rangle ^{-2s} \le C|h|^{2s-d}$ because 2s>d. Both constants stay bounded as long as d+2-2s and 2s-d stay bounded away from 0, which is the asserted uniformity in *s*.

*Upper bound, case *(b)*.* If 2s=d+2 the far sum is unchanged and contributes $\mathcal {O}(|h|^{2})$, while the near sum becomes $|h|^2\sum _{|k|\le 1/|h|}\langle k\rangle ^{-d} \le C|h|^2\log (2/|h|)$; adding the two gives the claim, and $\log (2/|h|) = \mathcal {O}(|h|^{-2(1-\epsilon )})$ for every ϵ<1.

*Lower bound, case *(a)*.* Fix $h_0:= \min \bigl (\tfrac{1}{4},\tfrac{1}{8k_1},h_1\bigr )$, where $h_1$ is chosen below, and assume first $0<|h| \le h_0$. Let

$$ A_h \;:=\; \Bigl \{k \in \mathbb {Z}^d \;:\; \tfrac{1}{8|h|} \le |k| \le \tfrac{1}{4|h|}\Bigr \}. $$

Choosing $h_1$ small enough that the inner radius exceeds an absolute constant, the lattice-point count in this annulus satisfies $\#A_h \ge c_2|h|^{-d}$, and every $k \in A_h$ has $\langle k\rangle |h| \in [c_4,C_4]$ with $c_4,C_4$ absolute, so that $\langle k\rangle ^{-2s} \ge c_3|h|^{2s}$ with $c_3 = \min (c_4,C_4)^{-2s}\wedge \max (c_4,C_4)^{-2s}$ bounded below uniformly for bounded *s*; and, since $|k| \ge 1/(8|h|) \ge k_1$, also $w(k) \ge c_1$. For $k \in A_h$ we have $|2\pi k\cdot h| \le 2\pi |k||h| \le \pi /2$, so the elementary inequality $1-\cos t \ge \tfrac{2}{\pi ^2}t^2$ for $|t| \le \pi$ gives $1-\cos 2\pi k\cdot h \ge 8\,(k\cdot h)^2$. Hence

$$ S(h) \;\ge \; 8\,c_1c_3\,|h|^{2\,s} \sum _{k \in A_h}(k\cdot h)^2 \;=\; 8\,c_1c_3\,|h|^{2\,s}\; h^{\top }\!M_h\,h, \qquad M_h:= \sum _{k\in A_h}k\,k^{\top }. $$

The set $A_h$ is defined by |*k*| alone, so it is invariant under every signed permutation of the coordinates. Writing *P* for the orthogonal matrix of such a transformation, $PA_h = A_h$ and therefore $PM_hP^{\top } = M_h$. A matrix commuting with all sign changes has vanishing off-diagonal entries, and one commuting with all coordinate permutations has equal diagonal entries; hence $M_h = \tfrac{1}{d}\operatorname {tr}(M_h)\,I = \tfrac{1}{d}\bigl (\sum _{k\in A_h}|k|^2\bigr )I$ exactly. Consequently

$$ h^{\top }M_h\,h \;=\; \frac{|h|^2}{d}\sum _{k\in A_h}|k|^2 \;\ge \; \frac{|h|^2}{d}\cdot \frac{1}{64|h|^2}\cdot c_2|h|^{-d} \;=\; \frac{c_2}{64d}\,|h|^{-d}, $$

and $S(h) \ge c\,|h|^{2s-d}$ as claimed.

For $h_0 \le |h| \le \tfrac{1}{4}$ the sum *S* converges uniformly, since $S(h) \le 2C_1\sum _k\langle k\rangle ^{-2s} < \infty$, and is therefore continuous; it is also strictly positive there, for S(h)=0 would force $k\cdot h \in \mathbb {Z}$ for every *k* with $|k| \ge k_1$ and hence $h \in \mathbb {Z}^d$, which is impossible for $0<|h|\le \frac{1}{4}$ on $\mathbb {T}^d$. A continuous positive function on the compact set $\{h_0 \le |h| \le \frac{1}{4}\}$ is bounded below, and $|h|^{2s-d}$ is bounded above there, so the stated inequality persists after enlarging the constant. Both bounds are joint continuous functions of (*s*, *h*) on the compact set obtained by letting *s* range over a compact subinterval of $(\tfrac{d}{2},\tfrac{d+2}{2})$, which gives the uniformity in *s*. □

### Periodisation and truncation

The covariances used in this paper are periodisations of Euclidean ones, and two of the arguments above rely on the fact that periodisation is invisible near the diagonal.

Throughout, $k^{\mathbb {R}}_\nu$ is the Euclidean Matérn kernel of Lemma A.14 and

A6 $$\begin{aligned} k_\nu (h) \;=\; \sum _{n \in \mathbb {Z}^d} k^{\mathbb {R}}_\nu \bigl (\Vert h+n\Vert \bigr ), \qquad h \in \mathbb {T}^d , \end{aligned}$$

is its periodisation, the covariance (11).

#### Proposition A.18

(Periodisation is smooth near the diagonal) Let $R_\nu (h):= k_\nu (h) - k^{\mathbb {R}}_\nu (\Vert h\Vert )$ denote the sum of the off-lattice terms in (A6). Then $R_\nu \in C^\infty$ on $\{\Vert h\Vert _\infty < \tfrac{1}{4}\}$, and for every multi-index α there is $C_\alpha = C_\alpha (\nu ,\ell ,\sigma ,d)$ with

$$ \sup _{\Vert h\Vert _\infty \le \tfrac{1}{4}}\bigl |\partial ^\alpha R_\nu (h)\bigr | \;\le \; C_\alpha \, e^{-\kappa /(2\sqrt{d})}. $$

The same bounds hold on a complex neighbourhood of $\{\Vert h\Vert _\infty <\tfrac{1}{4}\}$, so $R_\nu$ is real-analytic there. These are the bounds the increment comparison in the proof of Proposition 3.4 uses to carry Proposition 3.3 over to the torus.

#### Proof

Let $\Vert h\Vert _\infty \le \tfrac{1}{4}$ and $n \in \mathbb {Z}^d{\setminus }\{0\}$, so $\Vert n\Vert _\infty \ge 1$. Then $\Vert h+n\Vert _\infty \ge \Vert n\Vert _\infty - \tfrac{1}{4} \ge \tfrac{3}{4}\Vert n\Vert _\infty$, whence

A7 $$\begin{aligned} \Vert h+n\Vert \;\ge \; \Vert h+n\Vert _\infty \;\ge \; \tfrac{3}{4}\Vert n\Vert _\infty \;\ge \; \tfrac{3}{4\sqrt{d}}\,\Vert n\Vert \;\ge \; \tfrac{3}{4\sqrt{d}} . \end{aligned}$$

In particular no term with n≠0 has its singularity in the region considered, and $h \mapsto k^{\mathbb {R}}_\nu (\Vert h+n\Vert )$ is smooth there. Differentiating |α| times produces derivatives of $k^{\mathbb {R}}_\nu$ of order at most |α| multiplied by derivatives of $h \mapsto \Vert h+n\Vert$, which are bounded uniformly by (A7); Lemma A.14 and (A7) then give

$$ \bigl |\partial ^\alpha _h\, k^{\mathbb {R}}_\nu (\Vert h+n\Vert )\bigr | \;\le \; C_\alpha ' \, \Vert n\Vert ^{\nu -1/2}\, e^{-3\kappa \Vert n\Vert /(4\sqrt{d})}. $$

Splitting the exponential as $e^{-3\kappa \Vert n\Vert /(4\sqrt{d})} = e^{-\kappa \Vert n\Vert /(2\sqrt{d})}\, e^{-\kappa \Vert n\Vert /(4\sqrt{d})} \le e^{-\kappa /(2\sqrt{d})}\,e^{-\kappa \Vert n\Vert /(4\sqrt{d})}$, which uses $\Vert n\Vert \ge 1$, and summing the convergent series, $\sum _{n\ne 0}\Vert n\Vert ^{\nu -1/2}e^{-\kappa \Vert n\Vert /(4\sqrt{d})}$ gives the stated bound after relabelling constants. Uniform convergence of the differentiated series on the region gives $R_\nu \in C^\infty$. For analyticity, $z \mapsto z^\nu K_\nu (z)$ is holomorphic on ℜz>0 and the same estimates hold with $\Vert h+n\Vert$ replaced by its holomorphic extension on a complex neighbourhood on which (A7) persists, so the series converges uniformly there and $R_\nu$ is the restriction of a holomorphic function. □

#### Corollary A.19

(Truncation error) Truncating (A6) at $\Vert n\Vert _\infty \le N$ leaves an error

$$ \sup _{h\in \mathbb {T}^d}\Bigl |k_\nu (h) - \sum _{\Vert n\Vert _\infty \le N} k^{\mathbb {R}}_\nu (\Vert h+n\Vert )\Bigr | \;\le \; C\,N^{\,d-1+\nu -1/2}\, e^{-3\kappa N/(4\sqrt{d})} $$

for N≥1, with *C* depending on ν, ℓ, σ and *d* only. The same bound with $C_\alpha$ in place of *C* holds for each derivative $\partial ^\alpha k_\nu$.

#### Proof

By (A7) and Lemma A.14 each term with $\Vert n\Vert _\infty = M$ is at most

$$ C\,M^{\nu -1/2}\,e^{-3\kappa M/(4\sqrt{d})}, $$

and there are $O(M^{d-1})$ such lattice points. Summing the resulting series over M>N and bounding it by its first term times a convergent geometric factor gives the claim. □

### Proof of proposition 3.4

#### Proof of Proposition 3.4

Write $\rho _Z(x-x') = k_\nu (x,x')$, so that the stationary covariance (11) defines the operator $L_{\rho _Z}f = \rho _Z *f$ on $C^\infty (\mathbb {T}^d)$. Set

$$ j \;:=\; \lceil \nu \rceil -1, \qquad r \;:=\; 2\nu -2j \in (0,2], $$

so that ν=j+r/2, with r<2 exactly when $\nu \notin \mathbb {N}$.

By Poisson summation the Fourier coefficients of a periodisation are the samples at integer frequencies of the Euclidean Fourier transform of the summand: the identity $\sum _{n \in \mathbb {Z}^d} f(h+n) = \sum _{k \in \mathbb {Z}^d} \hat{f}(k)e^{2\pi i k\cdot h}$ holds pointwise, with both series absolutely convergent, as soon as $|f(x)| \le C(1+|x|)^{-d-\epsilon }$ and $|\hat{f}(\xi )| \le C(1+|\xi |)^{-d-\epsilon }$ for some ϵ>0. Both hypotheses hold for the Euclidean Matérn kernel $k^{\mathbb {R}}_\nu$ of Lemma A.14: that lemma gives exponential decay of $k^{\mathbb {R}}_\nu$ itself, and its transform is the Whittle–Matérn spectral density of [4],

A8 $$\begin{aligned} \widehat{k^{\mathbb {R}}_\nu }(\xi ) \;=\; c_1\Bigl (\tfrac{2\nu }{\ell ^2} + 4\pi ^2|\xi |^2\Bigr )^{-(\nu + d/2)}, \qquad c_1 = c_1(\nu ,\ell ,\sigma ,d) > 0 , \end{aligned}$$

which is $\mathcal {O}(|\xi |^{-2\nu -d})$ and therefore integrable precisely because ν>0.

Convolution on $\mathbb {T}^d$ is a Fourier multiplier, $\widehat{\rho *f}(k) = \hat{\rho }(k)\hat{f}(k)$, and the previous paragraph identifies $\hat{\rho }_Z(k) = \widehat{k^{\mathbb {R}}_\nu }(k)$. Since $\kappa ^2 = 2\nu /\ell ^2$, the operator $L_{\rho _Z}$ is therefore a Fourier multiplier with symbol

A9 $$\begin{aligned} \sigma (k) \;=\; c_1\bigl (\kappa ^2 + 4\pi ^2|k|^2\bigr )^{-(\nu +d/2)} \;=\; c_1\,\lambda (k)^{-2s}, \qquad s := \nu + \tfrac{d}{2} , \end{aligned}$$

of order m=-2ν-d.

Lemma A.16 applied with this *s* gives

$$ \bigl |\partial ^\alpha \rho _Z(h) - \partial ^\alpha \rho _Z(0)\bigr | = \mathcal {O}(\Vert h\Vert ^{2\epsilon }), \qquad |\alpha | = 2j, \quad \epsilon < r/2, $$

so the stationary criterion (10) holds with n=j and γ=r/2. By the “if” half of Theorem 3.2, samples lie in $C^{(j+r/2)^-}_{loc} = C^{\nu ^-}_{loc}$.

Proposition A.18 writes $k_\nu = k^{\mathbb {R}}_\nu + R_\nu$ with $R_\nu$ real-analytic on $\{\Vert h\Vert _\infty <\tfrac{1}{4}\}$, and Lemma A.15 expands the Euclidean kernel near the origin as

A10 $$\begin{aligned} k^{\mathbb {R}}_\nu (h) \;=\; A\bigl (\Vert h\Vert ^2\bigr ) \;+\; {\left\{ \begin{array}{ll} c_\nu \,\Vert h\Vert ^{2\nu }\,B\bigl (\Vert h\Vert ^2\bigr ), &  \nu \notin \mathbb {N}, \\ c_\nu \,\Vert h\Vert ^{2\nu }\log \Vert h\Vert \;B\bigl (\Vert h\Vert ^2\bigr ), &  \nu \in \mathbb {N}, \end{array}\right. } \end{aligned}$$

with *A* and *B* entire, B(0)=1 and $c_\nu \ne 0$; the logarithm in the second line is the one carried by $K_\nu$ at integer order.

Fix $\alpha := 2j\,e_1$, so |α|=2j, and take $h = t\,e_1$ with t↓0. The contributions of $A(\Vert h\Vert ^2)$ and of $R_\nu$ to $\partial ^{\alpha }\rho _Z(h) - \partial ^{\alpha }\rho _Z(0)$ are $\mathcal {O}(t^2)$: both functions are even and $C^\infty$ near the origin, so $\partial ^\alpha$ of each is again even and $C^\infty$ there, hence has vanishing gradient at 0, and Taylor’s theorem applies on the segment from 0 to *h*, which lies in $\{\Vert h\Vert _\infty <\tfrac{1}{4}\}$. By Proposition A.18,

A11 $$\begin{aligned} \bigl |\partial ^\alpha R_\nu (h) - \partial ^\alpha R_\nu (0)\bigr | \;\le \; C'_\alpha \, e^{-\kappa /(2\sqrt{d})}\,\Vert h\Vert ^2 , \qquad \Vert h\Vert _\infty \le \tfrac{1}{4} , \end{aligned}$$

with $C'_\alpha$ depending on α and on $\nu ,\ell ,\sigma ,d$ (Taylor’s remainder uses the bounds of Proposition A.18 at order |α|+2). For the singular term, $\partial _1^{2j}|t|^{2\nu } = \gamma _\nu \,|t|^{\,r}$ with

$$ \gamma _\nu \;=\; 2\nu (2\nu -1)\cdots (2\nu -2j+1) \;\ne \; 0, $$

the factors being strictly positive because 2ν>2j; the corrections supplied by *B* are $\mathcal {O}(|t|^{\,r+2})$. Hence

A12 $$\begin{aligned} \partial ^{\alpha }\rho _Z(t e_1) - \partial ^{\alpha }\rho _Z(0) \;=\; {\left\{ \begin{array}{ll} c_\nu \gamma _\nu \,|t|^{\,r}\bigl (1+\mathcal {O}(t^2)\bigr ) + \mathcal {O}(t^2), &  \nu \notin \mathbb {N}, \\ c_\nu \gamma '_\nu \,t^{2}\log (1/|t|)\bigl (1+o(1)\bigr ), &  \nu \in \mathbb {N}, \end{array}\right. } \end{aligned}$$

with $\gamma '_\nu \ne 0$. When $\nu \notin \mathbb {N}$ we have r<2, so the first line is of exact order $|t|^{\,r}$; when $\nu \in \mathbb {N}$ the logarithm dominates every $\mathcal {O}(t^2)$ contribution.

Let $\nu ' > \nu$ and suppose samples lay in $C^{\nu '^-}_{loc}$; write $\nu ' = n' + \gamma '$ with $\gamma ' \in (0,1]$.

If $\nu \notin \mathbb {N}$, then ν=j+r/2 with r/2∈(0,1), and since $C^{\nu '^-}_{loc} \subseteq C^{\nu ''^-}_{loc}$ for $\nu \le \nu '' \le \nu '$ we may take $\nu '' = j+\gamma ''$ with $\gamma '' \in \bigl (r/2,\min (1,\nu '-j)\bigr )$. Choosing $\epsilon \in (r/2,\gamma '')$, the “only if” half of Theorem 3.2 would force $|\partial ^\alpha \rho _Z(h)-\partial ^\alpha \rho _Z(0)| = \mathcal {O}(\Vert h\Vert ^{2\epsilon })$ with 2ϵ>r, contradicting the first line of (A12).

If $$\nu \in \mathbb {N}$$, then r=2 and j=ν-1, so $$\gamma ' \le 1$$ forces $$n' \ge \nu $$. The criterion at order $n^{\prime }$ requires $$\rho _Z \in C^{2n'} \subseteq C^{2\nu }$$. But differentiating (A10) 2ν times in the direction $$e_1$$ produces a term $$c_\nu \gamma ''_\nu \log |t|$$ with $$\gamma ''_\nu \ne 0$$, which is unbounded as t→0, while every other contribution stays bounded; hence $$\rho _Z \notin C^{2\nu }(\mathbb {T}^d)$$ and the criterion is not applicable at order $n^{\prime }$.

Either way the assumption fails, and the samples lie in $C^{\nu ^-}_{loc}$ and no more. □
